# Insights into the Role of the Berry-Specific Ethylene Responsive Factor *VviERF045*

**DOI:** 10.3389/fpls.2016.01793

**Published:** 2016-12-09

**Authors:** Carmen Leida, Antonio Dal Rì, Lorenza Dalla Costa, Maria D. Gómez, Valerio Pompili, Paolo Sonego, Kristof Engelen, Domenico Masuero, Gabino Ríos, Claudio Moser

**Affiliations:** ^1^Genomics and Biology of Fruit Crops Department, Research and Innovation Center, Fondazione Edmund MachSan Michele all’Adige, Italy; ^2^Instituto de Biología Molecular y Celular de Plantas, Universidad Politécnica de Valencia-Consejo Superior de Investigaciones CientíficasValencia, Spain; ^3^Computational Biology Department, Research and Innovation Center, Fondazione Edmund MachTrento, Italy; ^4^Food Quality and Nutrition Department, Research and Innovation Centre, Fondazione Edmund MachTrento, Italy; ^5^Fruit Tree Breeding Department, Instituto Valenciano de Investigaciones AgrariasMoncada, Spain

**Keywords:** ERF, RNA-seq, over-expressing transgenic lines, VOCs, wax, *Vitis vinifera*

## Abstract

During grape ripening, numerous transcriptional and metabolic changes are required in order to obtain colored, sweet, and flavored berries. There is evidence that ethylene, together with other signals, plays an important role in triggering the onset of ripening. Here, we report the functional characterization of a berry-specific Ethylene Responsive Factor (ERF), *VviERF045*, which is induced just before véraison and peaks at ripening. Phylogenetic analysis revealed it is close to the SHINE clade of ERFs, factors involved in the regulation of wax biosynthesis and cuticle morphology. Transgenic grapevines lines overexpressing *VviERF045* were obtained, *in vitro* propagated, phenotypically characterized, and analyzed for the content of specific classes of metabolites. The effect of *VviERF045* was correlated with the level of transgene expression, with high-expressing lines showing stunted growth, discolored and smaller leaves, and a lower level of chlorophylls and carotenoids. One line with intermediate expression, L15, was characterized at the transcriptomic level and showed 573 differentially expressed genes compared to wild type plants. Microscopy and gene expression analyses point toward a major role of *VviERF045* in epidermis patterning by acting on waxes and cuticle. They also indicate that *VviERF045* affects phenolic secondary metabolism and induces a reaction resembling a plant immune response with modulation of receptor like-kinases and pathogen related genes. These results suggest also a possible role of this transcription factor in berry ripening, likely related to changes in epidermis and cuticle of the berry, cell expansion, a decrease in photosynthetic capacity, and the activation of several defense related genes as well as from the phenylpropanoid metabolism. All these processes occur in the berry during ripening.

## Introduction

Fruit ripening is a developmental process whereby mature seed-bearing organs undergo physiological and metabolic changes that promote seed dispersal. These changes affect the nutritional value of fruit and are thus of key relevance for human and animal diet, but it also makes the fruits more susceptible to pathogen attacks, reasons for which the process attracts considerable attention from the scientific community (Giovannoni, 2004).

Grapevine is one of the most important cultivated crops in the world; the fruit is used as a source of fresh fruit, or once fermented, for production of wine and distilled beverages. The beginning of grape ripening, called véraison, coincides with a dramatic metabolic re-arrangement, affecting the accumulation of sugars, metabolism of acids, berry softening and coloring, and fruit growth. Ripening control in non-climacteric fruits, such as grapes, was originally thought to be ethylene independent, but recent evidence demonstrates a common genetic regulatory mechanism between climacteric and non-climacteric fruits (Lin et al., 2009). For example, a small amount of ethylene was measured in non-climacteric strawberries and this production was correlated to the expression of an ACC oxidase 1 gene (Trainotti et al., 2005). Other evidence includes the observation that climacteric, such as tomato, and non-climacteric species, such as grapevine, share common ripening regulators like members of the MADS-box, Zn-fingers, and bZIP transcription factor (TF) families (Fei et al., 2004).

There are hints suggesting that ethylene is also affecting grape ripening. The application on grapes of the ethylene releasing compound 2-chloroethylphosphonic acid (CEPA) 3–6 weeks before véraison causes a delay of the ripening process, while treatments 2 weeks before véraison accelerate the start of grape ripening (Coombe and Hale, 1973). Application of the inhibitor of the ethylene receptor 1-methylciclopropene (1-MCP) before véraison delays berry growth, acid degradation, sucrose production, and coloring (Chervin et al., 2004). A peak of endogenous ethylene has also been detected in grapevine berries, although at much lower concentrations than in climacteric fruits, 1 week before véraison (Chervin et al., 2004). The potential role of ethylene in the ripening of non-climacteric fruits is likely to occur via cross-talk with other hormones such as abscisic acid, auxin and brassinosteroids, all of which are known to play a part in grapevine berry ripening (Hale et al., 1970; Coombe and Hale, 1973; Davies et al., 1997; Jeong et al., 2004; Symons et al., 2006).

A key step in ethylene signal transduction is the activation of ethylene responsive factors (ERFs) that belong to the large superfamily of AP2/ERF TFs, specific to plants (Nakano et al., 2006). These factors are characterized by the presence of one or more AP2/ERF domains, consisting of 58–59 amino acids folded in one α-helix and a β-sheet, that recognizes the GCC box (5′-AGCCGCC-3′) *cis*-element in the promoter of the target genes (Fujimoto et al., 2000). Based on the number of AP2/ERF domains and presence of other conserved domains, this superfamily can be divided into three families called AP2, ERF and RAV. The ERF family is characterized by one single AP2/ERF domain and it comprises the CBF/DREB and ERF *sensu stricto* subfamilies (Sakuma et al., 2002). ERF members have been discovered in many plant species due to the high degree of conservation of AP2/ERF domain (Nakano et al., 2006; Zhang et al., 2008; Zhuang et al., 2008), including grapevine, where 132 and 149 AP2/ERF genes have been predicted (Zhuang et al., 2009; Licausi et al., 2010). ERF and DREB factors are often involved in fruit ripening control, and plant response to stress (Nakano et al., 2006). Among ERF proteins involved in fruit ripening are factors characterized in plum, apple and tomato. Seven ERFs have been proposed to regulate plum fruit development and ripening, based on their gene expression patterns (El-Sharkawy et al., 2009). *MdERF1* and *MdERF2* are regulated by ethylene in apple as suggested by exogenous MCP treatment and varietal studies (Wang et al., 2007). Overexpression and silencing of the tomato *LeERF1* gene has revealed an important role in plant development, fruit ripening and softening (Li et al., 2007), and tolerance to drought (Lu et al., 2010). Members of the *SHINE* clade of ERF factors (Aharoni et al., 2004) are involved in the regulation of lipid biosynthesis and the accumulation of cuticular waxes in tomato, leading to drought tolerance and recovery from water deficit (Shi et al., 2013).

In this study we focus on *VviERF045*, a factor phylogenetically related to the *SHINE* clade of *ERF* genes from *Arabidopsis* (Aharoni et al., 2004) which is specifically induced after véraison in grapevine fruit, and thought to play a role in the ripening process (Pilati et al., 2007; Fasoli et al., 2012; Lijavetzky et al., 2012; Palumbo et al., 2014). Five transgenic lines overexpressing *VviERF045* were obtained and used for functional characterization through phenotypic observation and metabolic and transcriptomic analyses.

## Materials and Methods

### Plant Material, 1-MCP and Etephon Treatments

Fruits were harvested from ‘Pinot Noir’ grapevine 10-years old plants cultivated in open field at Fondazione Edmund Mach (FEM) in San Michele all’Adige (Italy), following standard cultural practices and disease management. During 2006, three independent clusters were collected weekly starting from 4 to 10 weeks after anthesis (WAA) and at 14 WAA. Seeds, buds, tendrils, adult and young leaves, roots and flowers were also collected. The fruit (10 WAA) was dissected into pulp, skin and seed.

1-MCP and etephon treatments (both at 5 ppm) were performed at 7, 8, 9 WAA for 24 h, in a polyethylene bag wrapped around the cluster. Véraison (berry color change) occurred at 7 WAA. Mock treatments were applied to the control samples. Plant material was immediately frozen at -80°C and stored until analysis.

### Phylogenetic Analysis

**FIGURE 1:**
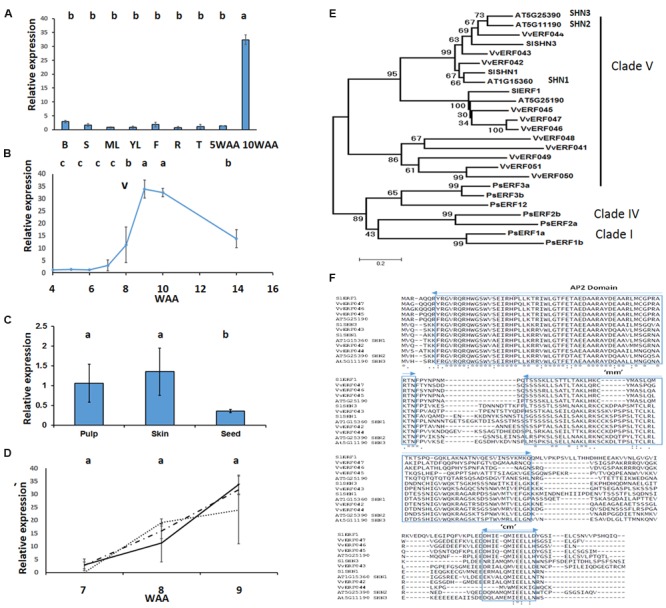
***VviERF045* expression pattern and protein sequence comparative analysis.** Left panel: real time RT-qPCR analysis of *VviERF045* expression profile in different tissues **(A)**: B, Bud; S, Shoot; ML, Mature Leaf; YL, Young Leaf; F, Flower; R, Root; T, Tendril; 5WAA, berry at 5 weeks after anthesis (WAA); 10 WAA, berry at 10 WAA. ML and YL were used as reference samples. **(B)** *VviERF045* expression in different fruit parts: pulp, skin and seed at 10 WAA. Pulp is taken as calibrator **(C)** *VviERF045* expression at different developmental stages, berry developmental stages are indicated as WAA, (v) indicates véraison. **(D)** *VviERF045* expression after 1-MCP and ethephon treatment: points represent 1-MCP treatment, points and lines represent ethephon treatment and continuous line represents control. Error bars represent SD and are based on three biological and two technical replicates. Data were normalized using ubiquitin and tubulin as reference genes. Different letters in the figure mean significant difference (*p* < 0.05) according to Tuckey’s *post hoc* test. Right panel: **(E)** Phylogenetic tree of the ERF amino acid sequences from *Prunus salicina* [PsERF1a (FJ026009), PsERF1b (FJ026008), PsERF12 (FJ026003), PsERF3a (FJ026005), PsERF3b (FJ026004), PsERF2a (FJ026007), PsERF2b (FJ026006)], *Arabidopsis thaliana* [AT5G25190 (NP_197901.1), AT1G15360-SHN1 (NP_172988.1), AT5G25390-SHN3 (NP_197921.1), AT5G11190-SHN2 (NP_196680.1)], *Solanum lycopersicum* [SlSHN1 (XP_004235965.1), SlSHN3 (XP_004240977.1), SlERF1 (NP_001234848.1)] and *Vitis vinifera* ERF from clade V [VviERF045, VviERF042, VviERF051, VviERF044, VviERF043, VviERF048, VviERF049, VviERF041, VviERF050, VviERF047, VviERF046]. The aa sequences were selected based on these criteria: (i) Grapevine ERFs belonging to the same clade of VviERF045, (ii) *Prunus salicina* ERF sequences related to fruit ripening, (iii) Best blastx matches to VviERF045 from *A. thaliana* and *S. lycopersicum*. Numbers on the branch represent the percentage for bootstrap value *n* = 1000. **(F)** Alignment of the amino acid sequences of clade V of the phylogenetic tree. ‘AP2,’‘mm’ and ‘cm’ conserved domains are represented as blue rectangles.

The protein sequences of VviERF045, 7 ERFs from *Prunus salicina* (El-Sharkawy et al., 2009) and the three best blastx matches to VviERF045 from *Solanum lycopersicum* and *Arabidopsis* were aligned with MUSCLE (Edgar, 2004). In order to assess the real orthologs, a reciprocal best hit approach was used. Genebank accession numbers are listed in **Figure 1F**. A distance matrix was constructed according to the PAM model and clustered with the Neighbor-Joining method, using the EMBL-EBI bioinformatic tools framework (Li et al., 2015). The reliability of the phylogenetic grouping was assessed by bootstrapping (1000 replicates).

### Production of Transgenic Lines

The complete coding region of *VviERF045* (GenBank accession number KX179904) was amplified with Pfu Ultra Hotstart DNA polymerase (Stratagene, San Diego, CA, USA), starting from cDNA from mature berry. The purified PCR product was cloned into pENTR-D TOPO cloning vector (Invitrogen, Carlsbad, CA, USA), sequenced and transferred to pK7WG2 binary vector (Karimi et al., 2002) downstream of the 35SCaMV promoter, by using the Gateway technology (Invitrogen). The *Agrobacterium* strain EHA105 containing the *VviERF045* binary vector and the pCH32 virulence helper plasmid were used for grape transformation. Gene transfer experiments were performed as described in Dalla Costa et al. (2016) on embyogenic calli of *Vitis vinifera* cv. ‘Brachetto’. Transgenic and wild type plants were grown and propagated *in vitro*.

### Expression by Quantitative Real-Time PCR (RT-qPCR) Analysis

Each sample was composed of a pool of leaves (first five leaves from the apical meristem) from five different *in vitro* plants. Total RNA was extracted from 100 mg of leaf powder by using SpectrumTM Plant Total RNA kit (Sigma–Aldrich, St Louis, MO, USA), adding 1% PVP40 in the extraction buffer. Total RNA was quantified with Nanodrop8000 Spectrophotometer (Thermo Scientific, Waltham, MA, USA). RNA integrity was checked by agarose gel electrophoresis. Total RNA (1 μg) was treated with Ambion^®^ DNA-free DNase Treatment in order to remove contaminating DNA (Life technologies, Carlsbad CA, USA), and subsequently reverse transcribed with SuperScript^®^VILO^TM^ cDNA Synthesis Kit (Invitrogen) in a final volume of 20 μL, according to manufacturer’s instructions. One microliter of a 10X diluted first strand cDNA was used for each amplification reaction in a final volume of 20 μL. RT-qPCR was performed in a ViiA^TM^ 7 Real-Time PCR System (Applied Biosystems, Foster City, CA, USA), using the KAPA SYBR Fast qPCR Master Mix (Kapa biosystems, Wilmington, MA, USA). Reaction composition and conditions followed manufacturer’s instructions. The cycling protocol consisted of 10 min at 95°C, then 40 cycles of 30 s at 95°C and 60 s at 60°C. Specificity of the PCR was assessed by the presence of a single peak in the dissociation curve after the amplification and through size estimation of the amplified product. The relative standard curve method was used to quantify relative expression genes in case of efficiency less than 90%. Otherwise the ΔCt method was used as described in Applied Biosystems user’s manual. Results were calculated as the average of three independent biological replicates for each line, repeated twice, using tubulin and ubiquitin as reference genes (Supplementary Table S2). For the amplification of *VviERF045*, we used two different primer pairs, namely “*VviERF045*” and “*VviERF045endog*” (Supplementary Table S2). Both primers of the first pair anneal on the coding sequence and they measure the total expression of the endogenous and exogenous (transgene) *VviERF045* copies. Unlike, the reverse primer of the second pair anneals on the 3′UTR region of the transcript which is present only in the endogenous gene copy but not in the transgenic one. The “*VviERF045endog*” primers were thus used to amplify specifically endogenous gene expression both in the transgenic lines and in the different grapevine tissues (**Figure 1**).

### RNA-Seq Analysis and Identification of Differentially Expressed Genes (DEGs)

Total RNA was extracted from three independent pools of leaves (1 g) from *in vitro* grown plants as described above. RNA-Seq for transgenic line L15 and control were performed using an Illumina HiSeq2000 sequencing service (Illumina, Inc., San Diego, CA, USA). Samples were sequenced twice in separated lanes. Paired-end (2 × 100 bp) and raw reads were pre-processed for quality using fastqc 0.11.2^1^ and adapter trimming with qtrim v0.94^2^. The resulting pre-processed reads were aligned to the reference transcriptome of *Vitis vinifera* (V1 grapevine annotation)^3^ using the bowtie2 aligner v2.2.3 (Langmead and Salzberg, 2012) and deposited in Gene Expression Omnibus^4^ series entry GSE77240. The summarized read count data was used to identify DEGs among various treatments by using the voom method (Law et al., 2014), which estimates the mean-variance relationship of the log-counts, generating a precision weight for each observation that is fed into the limma empirical Bayes analysis pipeline (Smyth et al., 2008). DEGs were identified between *OE_ERF* and *WT* using a *P*-value of 0.05 and a log2-fold change greater than 1.5 and lower than -1.5 (**Figure 4B**; Supplementary Table S4; **Supplementary Figure S1**).

### Functional Analysis

Differentially expressed genes were analyzed by BLAST2GOv 3.0.9 (Conesa et al., 2005) and TopGO (Alexa et al., 2006). The analysis with TopGO was done by comparing three statistical methods (Fisher’s, weight, Kolmogorov–Smirnov), and selecting the best 10 GO terms.

### Phenolic Metabolites Determination

Leaves from transgenic lines and control were sampled as described above (three biological replicates). Approximately 100 mg of powder from each sample was extracted in sealed glass vials using of a mixture of water/methanol/chlorophorm (20:40:40). Phenolics were extracted following Vrhovsek et al. (2012) method and UPLC chromatography was performed by injecting 2 μL of each sample. The same extract was used to measure anthocyanins by UPLC (Arapitsas et al., 2012).

### Lipid Profile Analysis

The lipid profile in leaves was determined following Della Corte et al. (2015) protocol, starting from 100 mg of powdered leaves and injecting 5 μL of lipid extracted solution into the LC-MS/MS system.

### Chlorophylls and Carotenoids Quantification

Leaves from *in vitro* cultivated transgenic lines and control were collected and powdered with liquid nitrogen (three biological replicates). Total carotenoids and chlorophylls were extracted from 100 mg samples using acetone 80% and read with a spectrophotometer at the wavelengths 470, 646.8, and 663.2 nm. Chlorophylls and carotenoids were determined following Lichtenthaler (1987) method.

### Leaf Area Measurement

Leaf area was measured with Iris Electronic Eye Analyzer VA300 (iBiosys Solutions Ltd, UK) and calculated with AlphaSoft 12.44 (Alpha MOS, France) using a fixed area as reference. In case of folded leaves the doubled part was cut and pasted aside with GIMP 2.6.12 image manipulation program (GNU GPL) in order to measure the whole leaf area. A non-parametric test was preferred for statistical analysis given the non-normality of data. We used the ggplot2 R package to graphically present these data in form of boxplots, using the geom_boxplot function (Wickham, 2009).

### PCA Analysis and Heatmaps

Principal component analysis (PCA) of the metabolites (**Supplementary Figure S4**) was obtained with R after scaling and centering the data. Heatmap representation of secondary metabolite content in the transgenic lines (**Figures 3**, **5**, and **6**) was calculated for each metabolite. Values were scaled by subtracting the mean value of WT and dividing by the standard deviation. Significance was assessed by ANOVA test followed by Tuckey’s test using R^5^.

### Optical Microscopy and CryoSEM

For lipid staining, a Sudan IV (Sigma–Aldrich) stock solution (0.1% w/v in isopropyl alcohol) was diluted 1:1 with glycerol, mixed well, and allowed to sit at room temperature for 30 min and syringe filtered to remove precipitates. The fourth or fifth leaves, starting from apex, were taken and cut in little squares of 4–5 mm. Leaves were included in agarose 5%, sectioned in 30 μm slices with a vibratome, stained for 30 min, mounted in distilled water with a cover slip and viewed immediately. Images were captured using a microscope Eclipse E600 (Nikon, Melville, NY, USA).

For Cryo-SEM, leaves were harvested, mounted on SEM stubs attached to a CT-1000C Cryo-transfer system (Oxford Instruments, Oxford, UK) and frozen in liquid N_2_. The frozen leaves were transferred to the cryo-stage of a JEOL JSM-5410 scanning electron microscope (SEM). The samples were then fractured, sublimated by controlled warming to -90°C, and sputter coated with a thin film of gold. Finally, leaves were viewed at an accelerating voltage of 15 keV and captured at 1000x and 2000x magnification.

## Results and Discussion

### *VviERF045* Is a Berry-Specific Transcription Factor Induced at Ripening and Closely Related to the ERFs from the SHINE-Clade

Expression analysis via RT-qPCR showed that *VviERF045* is highly expressed in fruit at 10 WAA, while its expression is much lower in other tissues such as root, stem, leaf, bud, flower and green berry (**Figure 1A**). During berry development *VviERF045* expression raises starting from 7 WAA and peaks 2–3 weeks later, about at the end of the véraison period, (**Figure 1B**) at which time, *VviERF045* expression is more pronounced in skin and pulp rather than seed (**Figure 1C**). These observations suggest that *VviERF045* might play a regulatory role in the berry ripening process. Although several members of the ethylene response factor family are ethylene inducible (Pirrello et al., 2012), berries treated around véraison (7, 8, and 9 WAA) with 1-MCP or etephon showed no significant differences in the expression of *VviERF045* compared with the control (**Figure 1D**). However, our study could not exclude that treatments done in a pre-véraison stage could have led to the same results.

Former phylogenetic analysis assigned VviERF045 to clade V of the ten clades identified for the 122 grapevine members of the ERF superfamily (Licausi et al., 2010). In this study we have made a more focused analysis comparing VviERF045 and other VviERFs from cladeV to 15 highly similar and previously characterized ERF protein sequences from *Prunus salicina, Arabidopsis thaliana*, and *Solanum lycopersicum* (**Figure 1E**). Our phylogenetic tree highlights that the seven ERFs related to fruit development and ripening in *Prunus salicina* (El-Sharkawy et al., 2009) cluster apart from the other ERF sequences. On the other side, VviERF045 and its homologs from tomato and *Arabidopsis* fall into a single clade together with VviERF046 and VviERF047. Interestingly, one subgroup into this clade contains exclusively wax biosynthesis genes whose overexpression results in a glossy leaf phenotype and increased drought tolerance: AT1G15360 (SHN1), AT5G11190 (SHN2), and AT5G25390 (SHN3) from *Arabidopsis*, and SlSHN1 and SlSHN3 from tomato (Tournier et al., 2003; Aharoni et al., 2004; Shi et al., 2013). They share a high degree of similarity since in addition to the conserved AP2 domain they display two other conserved motifs located in the middle and the C-terminus of the protein sequence (‘mm’ and ‘cm’; Aharoni et al., 2004). The remaining three sequences, which comprise VviERF045, SlERF1, and the *Arabidopsis* AT5G25190, form a distinct subgroup within clade V that distinguishes itself most notably by a deletion of six and one amino acid(s) in the ‘mm’ and ‘cm’ domains, respectively (**Figure 1F**).

AT5G25190 was reported to be induced by 1-aminocyclopropane-1-carboxylic acid (ACC) and salt (Zhang et al., 2011) as well as by drought (Huang et al., 2008), but it was shown that its overexpression does not lead to a typical leaf *shine* phenotype (Aharoni et al., 2004). *SlERF1* overexpression leads to several phenotypic effects including ethylene triple response on etiolated seedling, leaf development, enhanced fruit ripening and softening (Li et al., 2007) and improved tolerance to drought stress (Lu et al., 2010).

### Phenotypic Characterization of *VviERF045* Transgenic Lines

**FIGURE 2:**
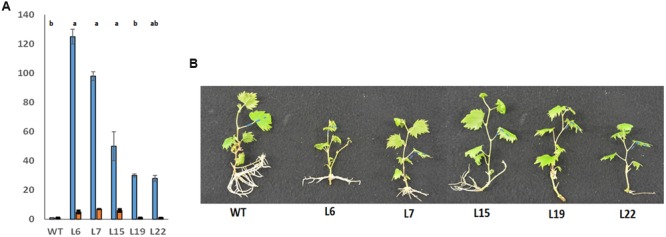
**Characterization of *VviERF045* overexpressing lines. (A)** Relative expression level of the endogenous + exogenous gene (blue bars), and endogenous gene (orange bars) in the transgenic lines. Error bars are based on data from three biological and two technical replicates. Data were normalized using ubiquitin and actin as reference genes. Relative expressions with the same letter are not significantly different (*p* < 0.05) according to Tuckey’s *post hoc* test. The ANOVA test refers to the red bars since for blue bars no significant difference was observed. **(B)** *In vitro* phenotype of transgenic and WT plants of the same age.

Fourteen transgenic lines overexpressing *VviERF045* from the *Vitis vinifera* cv. ‘Brachetto’ were generated (see Materials and Methods). In five of them, the expression of *VviERF045* (due to transcription of exogenous and endogenous gene copies) was much higher than in the wild type and they were used for further functional characterization. In lines L6 and L7 the expression of *VviERF045* was about 100-fold increased; in the other three lines it was increased around 25–30 times (**Figure 2A**).

**FIGURE 3:**
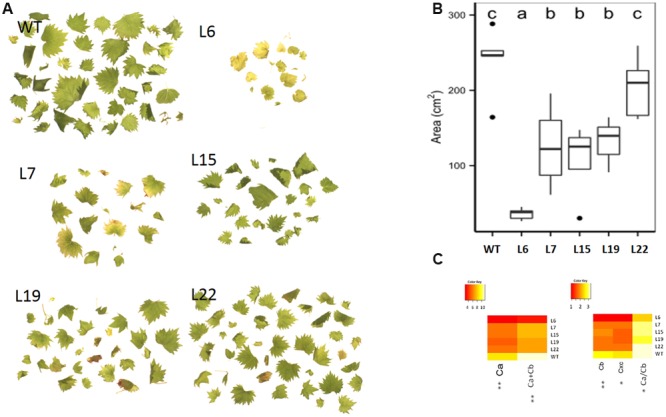
**Leaf area and color. (A)** Leaves morphology, **(B)** leaves total area measured in cm^2^. **(C)** Heatmap representation for chlorophylls and total carotenoids. (^∗^) and (^∗∗^) respectively indicates significant differences (*p* < 0.05), and highly significant differences (*p* < 0.01). A legend with the color scale is reported on the top left part of each heatmap.

The overall phenotype of the transgenic lines seemed directly related to the level of expression of the transgene, affecting not only leaf morphology and color, but also root biomass and architecture (**Figure 2B**) and this was particularly evident in case of L6. Several leaf features correlated strongly with the expression level of *VviERF045*, such as the leaf blade insertion angle on the petiole (**Figure 2B**), the leaf area and the leaf margins (**Figures 3A,B**). In general, L6 leaves displayed an acute insertion angle, a globular and chlorotic surface (almost yellow) and leaf margins curved toward the abaxial surface of the leaf, resembling somehow an ‘epinastic’ phenotype (Barry et al., 2001). Unlike L6, WT plants carried leaves with an insertion angle ranging between 180° and 140°, with a plane and bright green surface and an evident dentate margin. L15-19-22 showed an intermediate phenotype with insertion angles of 90–120°, a WT-like dentate leaf margin and a light green color, while L7 leaves were more similar to L6 in form and color. The analysis of pigment contents confirmed these phenotypic observations, with L6 showing lower values of chlorophyll a and b and carotenoids (**Figure 3C**). The transgenic lines showed also a general reduction in leaf number and leaf area. The total leaf area in L6 was about 10 times lower than in WT. L7, 15, and 19 showed comparable leaf areas, about half of the WT, whereas L22 was more similar to WT (**Figures 3A,B**). The *VviERF045* overexpressing lines had a smaller root system with short and thick roots (**Figure 2B**). This could be due to a defective auxin gradient, which plays a key role in root development (Overvoorde et al., 2010).

### Effect of *VviERF045* Overexpression on the Transcriptome

Three pools of leaves harvested from *in vitro* plants of the transgenic line L15 and from WT plants were used to compare the two transcriptomes by a RNA-Seq approach. L15 was selected since it showed a high level of *VviERF045* expression, while growing sufficiently well *in vitro* and *in vivo*. Between 29 and 79 million of paired-end reads of 100 nucleotides were obtained for each replicate, and on average 79% of them were properly aligned in both senses (Supplementary Table S1).

**FIGURE 4:**
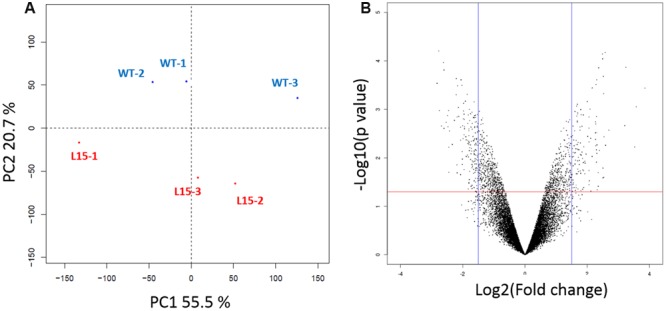
**RNA-Seq analysis of L15 line. (A)** Classical multidimensional scaling showing the percentage of variance explicated by PC1 and PC2. **(B)** Volcano plot used for selection of differentially expressed genes (DEGs) between L15 and WT lines. Spots represent genes. The red line represents the significance threshold of *p*-value (0.05) and the blue lines represent log2 fold change values of 1.5 and -1.5. Genes located above the red line and on the right-hand side or the left-hand side of blue lines were selected as DEGs.

A multidimensional scaling approach to the analysis of the expression data highlighted that the three replicates of L15 were well separated from those of WT (**Figure 4A**). Using a volcano plot, 573 DEGs between L15 and WT were identified in the region with absolute values of log2 fold change greater than 1.5 and a *p*-value <0.05 (**Figure 4B**; Supplementary Table S4).

To identify over-represented gene categories within the DEGs, we ran an enrichment analysis with both Blast2GO (Conesa et al., 2005) and TopGO (Alexa et al., 2006). Blast2GO found 35 categories (*p*-value < 0.05) from the ‘biological function’ subtree enriched with respect to the reference transcriptome (**Supplementary Figure S3A**). By grouping these GO categories into broader functional categories, phenylpropanoid metabolism, signaling and amino acid metabolism were over-represented. The analysis of GO terms using Blast2GO showed a higher percentage of transferase, protein phosphorylation, protein kinase and receptor activities, which suggests the participation of *VviERF045* in complex regulatory pathways. In addition, the presence of genes related to secondary metabolite pathways, such as trihydroxystilbene synthase, naringerin-chalcone synthase, flavonoid biosynthesis, and flavonoid metabolic process suggests the involvement of *VviERF045* in the synthesis and metabolism of phenolic compounds (**Supplementary Figure S3A**).

Nine major GO categories came out enriched by applying weight count versus classic count in Fisher’s enrichment test by using TopGO (Alexa et al., 2006): protein phosphorylation (Supplementary Table S4), wax biosynthetic process, response to endogenous stimulus, cotyledonal vascular tissue/pattern formation, drug transmembrane transport, jasmonic acid biosynthesis, response to salt stress (Supplementary Table S4), defense response to bacterium (Supplementary Table S4), and negative regulation of endopeptidase activity (**Supplementary Figure S3B**).

### Analysis of Metabolites

**FIGURE 5:**
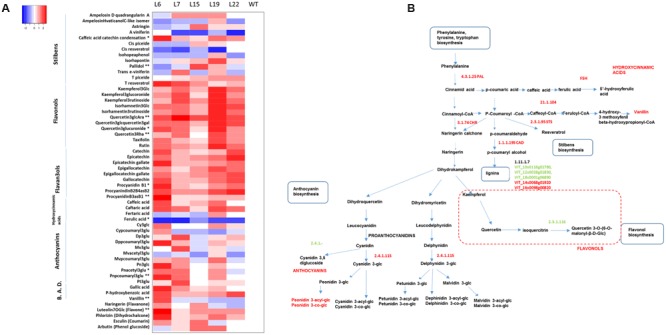
**Phenylpropanoid content in *VviERF045* overexpressing lines. (A)** Heatmap representation for phenylpropanoids content in the transgenic lines. For each metabolite, values are scaled by subtracting the WT mean value and dividing by the SD. (^∗^) and (^∗∗^) respectively indicates significant differences (*p* < 0.05), and highly significant differences (*p* < 0.01). A legend with the color scale is reported on the top left part. B.A.D., Benzoic acid derivative. **(B)** Transcripts involved in their biosynthesis. 4.3.1.25 Phenylalanine ammonium lyase (PAL) (*VIT_16s0039g01170, VIT_16s0039g01240, VIT_16s0039g01280, VIT_16s0039g01300, VIT_16s0039g01360*), 1.1.1.195 cinnamyl-alcohol dehydrogenase (CAD), (*VIT_03s0110g00310, VIT_13s0064g00270*), ferulate 5-hydroxylase (F5H) *(VIT_17s0000g03930*), 2.3.1.74 Chalcone reductase (CHR) (*VIT_01s0011g06440*), 21.1.104 Caffeoyl-CoA *O*-methyltransferase 1 (*VIT_07s0031g00350*), 1.11.1.7 peroxidase (*VIT_12s0028g01830, VIT_14s0068g01920, VIT_10s0116g01780, VIT_18s0001g06890, VIT_16s0098g00820*), 2.3.1.95 STS (stilbene synthase) (*VviSTS2, VviSTS3, VviSTS5, VviSTS6, VviSTS7, VviSTS10, VviSTS13, VviSTS15, VviSTS17, VviSTS18, VviSTS19, VviSTST20, VviSTST21, VviSTST25, VviSTS25, VviSTS28, VviSTS29, VviSTS30, VviSTS31, VviSTST37, VviSTST38, VviSTST39, VviSTST42, VviSTS46, VviSTS47* (Vannozzi et al., 2012). 2.3.1.116 Quercetin 3-*O*-glucoside-6″-*O*-malonyltransferase (*VIT_12s0134g00630*). 2.4.1.115 anthocyanidin 3-*O*-glucosyltransferase (*VIT_03s0017g02110, VIT_16s0022g01970*), 2.4.1- UDP-glucose (*VIT_18s0041g00830, VIT_18s0041g00840, VIT_18s0041g00930, VIT_18s0041g01010, VIT_16s0050g01680*). Green color means down-regulated gene, red color up-regulation.

**FIGURE 6:**
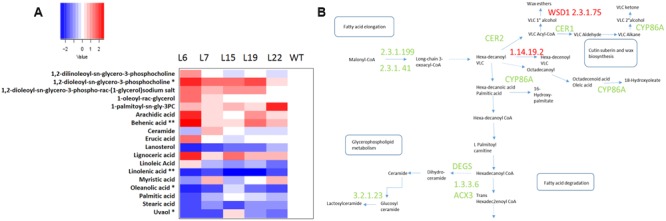
**Lipid content in *VviERF045* overexpressing lines. (A)** Heatmap representation for lipids content in the transgenic lines and WT. (^∗^) and (^∗∗^) indicates significant differences (*p* < 0.05), and highly significant differences (*p* < 0.01) for ANOVA and Tuckey’s tests, respectively. A legend with the color scale is reported on the top left part. **(B)** Transcripts involved in Fatty acid biosynthesis. 2.3.1.199 (LACS), 2.3.1.41 Beta-ketoacyl-CoA synthase (*VIT_00s0179g00380, VIT_16s0050g00830, VIT_01s0011g03490*), 1.14.19.2 (*VIT_02s0012g02500*) Acyl-[acyl-carrier-protein] desaturase, 1.3.3.6 Acyl-CoA oxidase (ACX3) (*VIT_02s0012g02920*), 3.2.1.23 beta-galactosidase (*VIT_00s0404g00010*), Sphingolipid delta 4 desaturase (DEGS) (*VIT_00s0583g00030*) Sphingolipid delta 4 desaturase DES-1, CYP86A (*VIT_02s0025g03320*), CYP86A (*VIT_07s0031g01680*), CER1 Eceriferum 1 Sterol desaturase (*VIT_15s0021g00050*), CER2 (*VIT_05s0029g00480*), CER3 (*VIT_08s0007g00390*), 2.3.1.75 WSD1 Wax Synthase desaturase 1 (*VIT_15s0046g00490*) (Yeats et al., 2014). Green color means down-regulated gene, red color means up-regulation.

We measured 56 phenolic secondary metabolites (**Supplementary Figure S4**), including chlorophyll a and b and total carotenoids (**Figure 3C**), hydroxycinnamic acids, benzoic acids, stilbenes, flavonols, flavan-3-ols and anthocyanins (**Figure 5A**) and lipids (**Figure 6A**) for the all five transgenic lines and WT (Supplementary Table S3). To have a general idea of the dispersion of the metabolic data, a PCA was computed for all the metabolites (**Supplementary Figure S4**). In this analysis the WT and the L6 plants were the most separated groups, whereas the other lines showed an intermediate position. Thus, the metabolomic study confirmed the extreme behavior of L6 line observed in the phenotypic characterization.

### *VviERF045* Is Involved in Plant Growth and Development

Among the DEGs we found a significant number of genes whose *Arabidopsis* putative orthologs are involved in growth and development, and more specifically are associated to the development of anatomical structures, the formation of cotyledonal vascular pattern, procambial histogenesis and multidimensional cell growth (Supplementary Table S4).

Noteworthy was *VIT_04s0008g01970*, coding for the putative ortholog of the *ERECTA* (*ER*) gene, which appeared strongly down-regulated in L15. *ER* codes for a leucine-rich repeat receptor-like Ser/Thr kinase that is a major transcriptional regulator with pleiotropic effects on development and plant physiology. It controls plant transpiration efficiency, modulating stomatal opening and CO_2_ fixation (Masle et al., 2005), stomatal density and patterning (Lampard et al., 2008), abaxial-adaxial identity (Qi et al., 2004), petal shape and size (Abraham et al., 2013), ethylene induced hyponastic growth and leaf petiole angle (Van Zanten et al., 2010), leaf area and plant biomass during shade avoidance syndrome (SAS) (Kasulin et al., 2013), and resistance against specific pathogens such as fungi (Häffner et al., 2014). Indeed *er* loss of function mutants show reduced plant size, rounder and shorter leaves, shorter petioles and compact inflorescences in *Arabidopsis*. These features closely resemble those we observed in the lines overexpressing *VviERF045*, namely reduced leaf biomass, leaves with globular surface and different leaf margins and changes in leaf-petiole angle (**Figure 2B**). In L15, *VIT_18s0001g10160*, coding for the putative *WUSCHEL-RELATED HOMEOBOX4* (*WOX4*) grapevine ortholog, was induced twofold with respect to WT plants. *WOX4* is expressed in the pro-cambium and plays an important role in vascular meristem organization. Recent evidence indicated that *ER* participates also in vascular development, acting upstream to *WOX4* (Uchida and Tasaka, 2013), and our results suggest a similar interplay between these genes in grapevine.

### *VviERF045* Regulates Lipid Metabolism As Well As Cuticle and Waxes Synthesis

**FIGURE 7:**
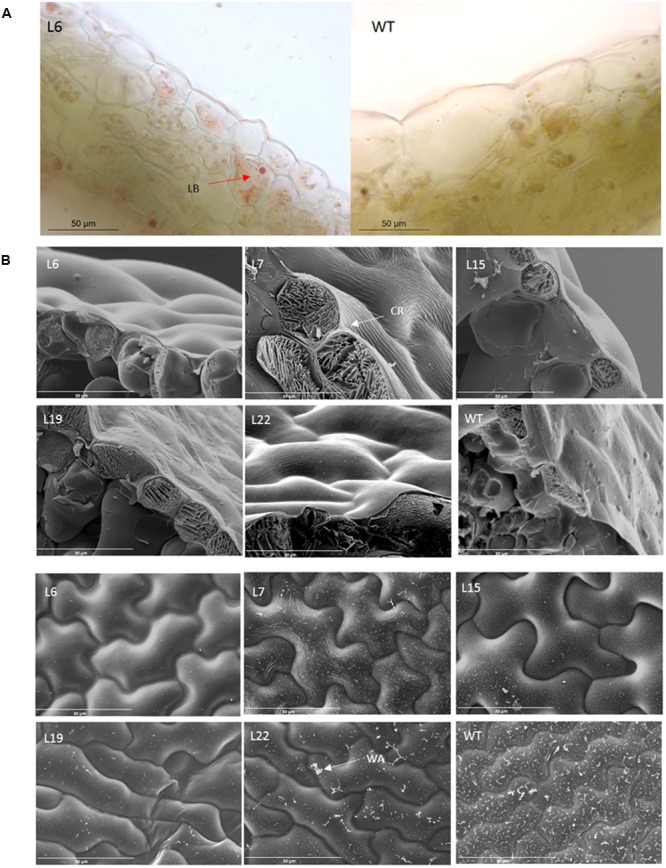
**Microscopic analysis of *VviERF045* overexpressing lines. (A)** Differences in lipids vesicle content in trangenic L6 and WT observed at 50X magnification and by SudanIV staining. LB, lipid bodies. **(B)** Changes in wax load and cuticle surface morphology of the transgenic lines detected by Scanning Electron Microscopy after freeze fracture (CryoSEM). Upper part: images of transversal section of the adaxial leaf surface. WT and L19 show wax deposition. (2000X) CR, Cuticular Ridge. Lower part: images of the adaxial leaf surface (1000X). WA, Wax Aggregates.

Our results show that *VviERF045* is functionally linked to lipid metabolism, specifically to the synthesis of cuticle and cuticular waxes. Optical images revealed a different pattern of the lipid distribution on the surface of L6 leaves compared to WT (**Figure 7A**). In the latter, the reddish color was evenly distributed along the cuticular layer that covers the epidermal cells, while in L6 the stain was observed in groups of intracellular droplets, similar to lipid bodies, in the epidermal layers. Scanning Electron Microscopy (Cryo-SEM) confirmed a striking difference in the structure of the epicuticular waxes between the two (**Figure 7B**): the WT cuticle appeared heavily decorated with wax aggregates, while the L6 leaf surface was smooth. The wax decoration in the other transgenic lines was reduced compared to WT.

**FIGURE 8:**
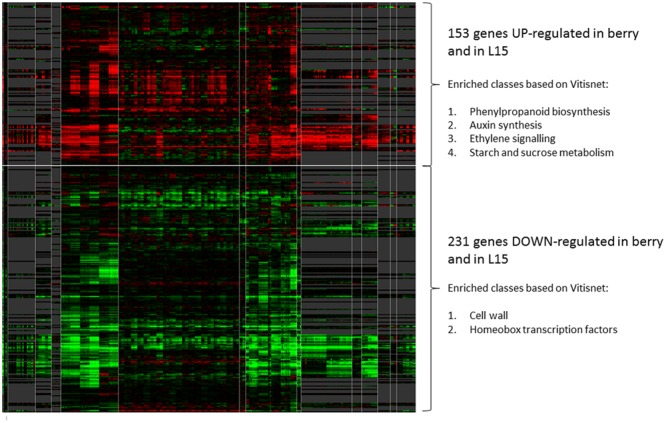
**Expression analysis of DEGs during berry ripening.** Heatmap showing L15-DE genes modulated during berry ripening in 389 condition contrasts derived from 16 experiments relative to berry samples at different developmental stages, between EL 27 and EL 41. The heatmap was produced using the grapevine gene expression compendium VESPUCCI (http://vespucci.colombos.fmach.it/). Genes up-regulated or down-regulated in ripe berry are grouped respectively in the upper or lower part of the heatmap. The white vertical lines delimit the different experiments to which the selected contrasts belong. On the right, some enriched categories (Vitisnet) within the up- or down-regulated genes are listed.

The lipid composition of leaf samples was analyzed to further understand the observed alterations at the cuticular level. Six lipid compounds belonging to the classes of fatty acids, sterols, glycerolipids, glycerophospholipids and sphingolipids appeared significantly modulated (**Figure 6A**). The steroid-like lanosterol was at a higher concentration in WT and diminished in transgenic lines proportionally to *VviERF045* expression (**Figure 6A**). Lanosterol is known to be a component of the tomato cuticular waxes. In the *lcer6* mutant, an increase of lanosterol together with other sterols and triterpenic cycles, was accompanied by a proportional decrease in long chain alkanes (Leide et al., 2007). This result is in line with the down-regulation in L15 of a squalene monoxigenase (*VIT_00s0441g00020*), involved in the oxidation of squalene to squalene epoxide, the precursor for lanosterol biosynthesis (**Figure 6B**).

Cuticular waxes are made of very-long-chain (VLC) fatty acids (FAs), synthesized starting from plastidial C16-C18 FAs, elongated into VLCFAs in the endoplasmic reticulum membrane, and subsequently modified into primary alcohols and wax esthers (**Figure 6A**, Yeats and Rose, 2013). Several genes involved in FA elongation and wax biosynthesis (*VIT_00s0179g00380, VIT_01s0011g03490, VIT_16s0050g00830*) were expressed at lower level in the L15 transgenic line (Supplementary Table S4), implying *VviERF045* overexpression reduces long FA and wax content. Down-regulation was observed for the putative orthologs of the *Arabidopsis CER1* and *CER2* genes, the grapevine genes *VIT_15s0021g00050* and *VIT_05s0029g00480*. The *cer1* mutant of *Arabidopsis* is blocked in the conversion of stem wax C30 aldehydes to C29 alkanes, leading to a lack of secondary alcohols and ketones. This biochemical impairment results in a reduced wax load on the leaf surface of the *cer1* mutants (Bourdenx et al., 2011), resembling the cuticular phenotype we observed in the transgenic lines in this study. The closest sequence to *VIT_05s0029g00480* is *CER26*, the homologue of *CER2*, which encodes for an acyl-transferase enzyme involved in the elongation process of C28 FAs (Pascal et al., 2013). *VIT_08s0007g00390*, similar to the *Arabidopsis PROTEOLYSIS 6* (*PRT6*), was less expressed in the L15 line. *prt6* mutants are impaired in lipid degradation and retain oil bodies in the cells, similar to the ones observed in the external layers of the L6 leaves in our analysis (**Supplementary Figure S6**) (Holman et al., 2009). The cytochrome P450 genes *VIT_02s0025g03320* and *VIT_07s0031g01680* were found down-regulated in *VviERF045* overexpressing lines. *VIT_02s0025g03320* belongs to the CYP86A subfamily, known to have ω-hydroxylase activity on midchain FAs (Yeats and Rose, 2013). The similar *Arabidopsis* gene *LACERATA* was reported to be involved in cutin biosynthesis (Wellesen et al., 2001). The most similar sequence to *VIT_07s0031g01680* in *Arabidopsis* belongs to the CYP96A subfamily, which includes MIDCHAIN ALKANE HYDROXYLASE 1 (MAH1, CYP96A15), an enzyme of the decarbonylation pathway catalyzing the synthesis of cuticular wax secondary alcohols and ketones from VLC alkanes (Greer et al., 2007). L15 plants also displayed lower expression of three lipases belonging to the GDSL family (*VIT_13s0106g00350, VIT_18s0041g02160, VIT_18s0086g00220*). Members of this large gene family appear to have a broad range of activities in the regulation of plant development, morphogenesis, synthesis of secondary metabolites, and defense response (Chepyshko et al., 2012). Recently, specific members within the family have been shown to play a role in cutin synthesis by catalyzing the formation of cutin ester oligomers (Yeats et al., 2014).

The only two genes of the wax biosynthetic pathway which resulted up-regulated in L15 were *VIT_02s0012g02500* and *VIT_15s0046g00490*, encoding for a putative stearoyl-acyl carrier protein-desaturase (S-ACP-DES) and a putative wax synthase/diacylglycerol acyltransferase 1 (WSD1), respectively (**Figure 6A**). In plants, S-ACP-DESs tune the ratio of saturated to monounsaturated FAs (Kachroo et al., 2007). In *Arabidopsis*, WSD1 is responsible for the esterification of VLC primary alcohols to long chain wax esthers using C16 FAs as substrates (Li et al., 2008).

As our results pointed toward a role of *VviERF045* in regulating cuticle biosynthesis, we compared the effects of its overexpression with those described for the major known regulators, namely the *SHINE* gene family and some specific *MYB* TFs. *WAX INDUCER1/SHINE1* (*WAX1/SHN1*) was the first TF identified (Aharoni et al., 2004). It is an ERF sequence of clade V, whose overexpression gives rise to dwarf plants with curved and glossy leaves, lower stomata density, thicker cutin and higher wax density. The cauline leaves of the gain of function mutant *shine* display cuticular ridges similar to those here reported on the L7 leaves (Aharoni et al., 2004; Kannangara et al., 2007). When *SHN1* and the other two closely related members *SHN2* and *SHN3* were silenced, *Arabidopsis* plants exhibited, among other phenotypic traits, a decrease in cutin load (Shi et al., 2011), and changes in cuticle structure and lipid composition have also been demonstrated in tomato (Shi et al., 2013). SHINE regulators exert their function by acting on several cuticle- and epidermis-associated genes, including *CYTOCHROME P450s, GSDL-type LIPASES, ACYLTRANSFERASES, LONG CHAIN ACYL_CoA SYNTHASES, CER1* and *CER2* (Kannangara et al., 2007; Shi et al., 2011, 2013). Genes with similar functions were down-regulated in L15 transgenic plants, as reported above.

AP2-containing TFs can be either activators or repressors depending on the effect on transcription of specific target genes. Transcriptional repressors are further classified as active or passive repressors: active repressors contain a repression domain (RD), which allows these proteins to actively prevent transcription of a target gene; passive repressors do not have an RD and suppress transcription by competing with transcriptional activators for binding to the target sequence (Licausi et al., 2013). *VviERF045* can not be classified as an active repressor because its sequence does not display a RD. The cuticular phenotype of the lines, as revealed by microscopical investigation, as well as the negative regulation of the cuticle- and wax-related genes in L15, are similar to those reported for *SHINE* silenced lines of *Arabidopsis* and tomato, indicating *VviERF045* as a potential passive repressor. In particular VviERF045 might negatively regulate *VviERF042* encoded by VIT_09s0002g06750 and VviERF044 encoded by VIT_04s0008g05440, which are down-regulated in L15 (Supplementary Table S4). Interestingly, *VviERF042* and *VviERF044* are the putative horthologues of the *Arabidopsis SHINE1* and *SHINE3* genes, whose silencing leads to a decrease in cutin load and to changes in cell wall structure (Shi et al., 2011) similar to the ones observed in the transgenic lines of this study.

The FA chain α-linolenic acid is also the precursor of the phytohormone Methyl Jasmonate (MeJA) via the action of a lipoxygenase and a jasmonate *O*-methyltransferase. In L15 we found up-regulated the genes encoding for these two enzymes *(VIT_06s0004g01470* and *VIT_14s0006g02170*), as well as for a MeJA esterase *(VIT_00s0253g00090)*, catalyzing the inverse reaction from MeJA to JA (**Supplementary Figure S6**). MeJA and JA are considered to be defense-related hormones and they do not seem to play a major role during berry ripening. It is not clear if the observed induction of the MeJA biosynthetic genes in L15 is related to the stress induced by the expression of the transgene or to direct regulatory effect of *VviERF045*.

### *VviERF045* Modulates Genes Involved in Secondary Metabolic Processes

Phenolics are a large and complex group of secondary metabolites with chemical properties that contribute to pigmentation and defense against several biotic and abiotic stresses in grapes (Ali et al., 2010). Their biosynthesis starts from the amino acid phenylalanine which is converted into a vast array of molecules belonging to the major classes of the phenylpropanoids (hydroxycinnamic acids, stilbenes and lignins) and of the flavonoids (flavonols, proanthocyanidins and anthocyanins) (**Figure 5B**).

Several DEGs belonging to the phenylpropanoid and flavonoid biosynthetic pathway (Supplementary Table S4) encode for enzymes often positioned at the branching point of the pathway. In agreement with the overall increases in phenolic compound concentration in the transgenic lines, the majority of related DEGs also were up-regulated (Supplementary Table S4, **Figure 5B**). This includes the induction of five *PHENYLALANINE AMMONIA-LYASE* (*PAL*) encoding genes (*VIT_16s0039g01170, VIT_16s0039g01240, VIT_16s0039g01280, VIT_16s0039g01300 and VIT_16s0039g01360*), which catalyze the conversion of L-phenylalanine to *trans*-cinnamic acid and ammonia. Among the DEGs there are also genes known to affect lignin amount and composition, suggesting that this metabolic class was likely induced as indicated by the high level of vanillin in L6 (Vanholme et al., 2010). In particular, different genes coding for cinnamyl-alcohol dehydrogenases (CADs), ferulate 5-hydroxylase (F5H), caffeoyl-CoA *o*-methyltransferase (COMT1) and several peroxidases were induced in L15 (**Figure 5B**). It is interesting to note that *SlSHN3* silenced tomato lines showed a thicker cell wall of the epidermal cells, and that Ambavaram et al. (2011) reported that *AtSHN2* controls secondary cell wall biosynthesis (lignin and cellulose) acting on *CAD* genes (Ambavaram et al., 2011), observations that support *VviERF045* acting as a *SHINE* factor.

Twenty-three stilbene synthase (STS) genes appeared positively associated to *VviERF045* over-expression. *STSs* form a rather expanded gene family in grapevine, including at least 33 members (Vannozzi et al., 2012), and they produce the basic stilbene structure, *trans*-resveratrol, from one *p*-coumaroyl-CoA and three malonyl-CoA molecules. *Trans*-resveratrol can then be modified by hydroxylation, methylation, glycosylation, or condensation of more units to form the ample class of stilbenoids, which represent the major antimicrobial phenolic compounds in grapevine (Jeandet et al., 2002; Malacarne et al., 2011). These compounds are also produced upon abiotic stresses such as UV-light, salinity stress (Ismail et al., 2012), and during leaf senescence and fruit ripening (Gatto et al., 2008).

The only highly accumulated stilbene common to all transgenic lines was the glucoside derivative of *t*-resveratrol, *trans*-piceide, but in L15 and L19 also the monomers *cis*-piceide, isorhapontin, astringin and the dimers pallidol and ampelopsin D exhibited higher levels than in WT plants. Since polymeric forms of resveratrol are usually produced during fungal attacks (Malacarne et al., 2011), this might indicates that *VviSTS* up-regulation in L15 was mainly driven by a more general stress (Cuendet et al., 2000). In grapes, flavan 3-ols are mainly present in skin and seed tissues, where they accumulate before véraison. In vegetative organs, their content constantly increases during leaf development, but their synthesis decreases in old leaves (Bogs et al., 2005). They are found as monomers, namely catechin, epicatechin and epicatechin 3-*O*-gallate, as well as oligomers, and polymers called proanthocyanidins (PA), also known as condensed tannins. In our transgenic lines, compounds of this class, either in monomeric or dimeric form (procyanidin B), or condensed to caffeic acid, were clearly found at higher concentration than in WT (**Figure 5A**). As flavan 3-ols appear to function in resistance against various biotic and abiotic stresses, including UV irradiation by decreasing oxidative stress (Hammerbacher et al., 2014), it is likely that the transgenic lines face a more stressful situation than WT plants, due, for example, to cuticle impairment and to reduced photosynthetic capacity.

In our experiment, a significant higher content of peonidin *p*-coumaryl3glu and to a lesser extent of the glycosylated forms of cyanidin, delphinidin and malvidin, were observed in most transgenic lines (**Figure 5A**). The glycosylated forms of the flavonols quercetin and isorhamnetin displayed a similar behavior. Where the main role of anthocyanins in grapes is the red berry pigmentation to attract animals for seed dispersal, the main function of flavonols is UV-protection. Both classes are antioxidant molecules induced during different stresses, which might be the main reason of their increase in the transgenic over-expression lines. In case of the anthocyanins, the expression data were congruent with the metabolic data for two anthocyanidin 3-*o*-glucosyltransferases up-regulated in L15 (*VIT_03s0017g02110, VIT_16s0022g01970*, Supplementary Table S4), but less coherent for five *MATE* genes (*VIT_11s0052g01560, VIT_11s0052g01540, VIT_07s0031g00750, VIT_00s0225g00080, VIT_11s0052g01500* Supplementary Table S4), which were down-regulated. This grapevine protein family plays a role in the H^+^-dependent transport of acylated anthocyanins into the vacuole (Gomez et al., 2009), and the observed down-regulation possibly indicates a problem with the vacuolar storage of these molecules.

Another important class of secondary metabolites affected in the transgenic over-expressing lines was the photosynthetic pigments, namely chlorophylls and carotenoids. As expected from the pale leaf color, the analysis of chlorophylls and carotenoids confirmed a much lower concentration in the transgenic lines, with a minimum in L6 (**Figure 3C**). During fruit ripening the photosynthetic apparatus is dismantled (Lijavetzky et al., 2012), and our results suggest that *VviERF045* might play such a role in the berries. In *Arabidopsis COP1-INTERACTING PROTEIN 7* (*CIP7*) is involved in light-dependent anthocyanin and chlorophyll accumulation (Yamamoto et al., 1998). The putative *CIP7* gene of grapevine (*VIT_00s1306g00010*) was down-regulated in our study, as confirmed by RT-qPCR (**Supplementary Figure S2**). This gene was reported to be down-regulated at véraison in five red Italian varieties (Palumbo et al., 2014), as well as during post harvest withering (Fasoli et al., 2012). Other L15 repressed genes related to chlorophyll metabolism are *FERRITINS* (*VIT_08s0058g00410, VIT_08s0058g00430, VIT_08s0058g00440*), iron-storage proteins involved in the regulation of free iron levels in the cells, whose impairment cause rapid natural senescence with leaf yellowing accompanied by accelerated decrease of maximal photochemical efficiency and chlorophyll degradation (Murgia et al., 2007).

In the transgenic lines, we observed the up-regulation of sesquiterpene synthase genes encoding for delta-cadinene synthase, alpha-farnesene synthase and valencene synthase (Lücker et al., 2004) (**Supplementary Figure S5**). Sesquiterpenes are a class of volatile terpenoids enriched in the epicuticular wax layer of the berry fruit. They act as antimicrobial volatile compounds (Petronilho et al., 2014) and they are induced by pathogenic fungi as well as by elicitors and MeJA (Hampel et al., 2005), but they contribute to the typical flavor of aromatic grape varieties too.

### *VviERF045* in Fruit Ripening

Berry ripening is a complex physiological process under tight regulation, which begins about 8 WAA and proceeds for about 5–6 weeks. From ripening onset, the berry undergoes chlorophyll degradation, accumulation of color, sugar and aroma compounds, organic acid catabolism, and an increase in berry size and elasticity (Coombe and McCARTHY, 2000).

Among the ERF regulatory factors possibly linked to the berry ripening process, identified previously in a microarray experiment on Pinot Noir berries at three developmental stages (Pilati et al., 2007), we selected *VviERF045* for further characterization, since this factor displays a fruit ripening specific expression (**Figure 1**). An important role for *VviERF045*, as major switch in berry ripening, was recently also proposed by Palumbo et al. (2014).

Although our study was not conducted on berries, but in leaves from *in vitro* plants, implying that the results cannot be transferred straightforwardly to the fruit system, we have observed the modulation of several processes in the transgenic overexpressing lines, which are also typical of grape ripening: changes in the epidermis and in the cuticle, a decrease in photosynthetic capacity, and the activation of several defense related genes.

In this study, we collected clear evidence that VviERF045 regulates wax biosynthesis and the morphology of the cuticle and probably of the cell wall in the epidermal cells by modulating a set of specific genes. The phylogenetic proximity of VviERF045 to the SHYNE clade (**Figure 1E**) of ERFs, known to function in cuticle and epidermis patterning, further corroborates this conclusion.

At ripening onset, three processes take place, all of which imply a modification of the outer structures of the epidermal cells and thus possibly the intervention of VviERF045: berry softening, berry expansion (Coombe, 1992), and a reduction in the thickness of cuticular waxes (Rogiers et al., 2004). In the overexpressing transgenic line L15, genes known to be involved in these berry processes, such as an endo-1,4-beta-glucanase (*VIT_04s0008g02010)* involved in cell wall disassembly (Libertini et al., 2004), three expansins (*VIT_06s0004g04860, VIT_06s0004g07970, VIT_12s0059g00190*), a polygalacturonase PG1 (*VIT_07s0005g01550)*, and a pectinesterase (*VIT_11s0016g00330*) related to berry expansion and skin softening (Deytieux-Belleau et al., 2008), are down-regulated compared to the WT plants. These same genes are induced in the berry, at ripening onset. This might suggest that *VviERF045* down-regulates these enzymes to counterbalance an excessive cell wall disassembling. The post-véraison development of an amorphous layer of cuticular waxes and the observation that deposition of epicuticular wax ceases at véraison as reported in Shiraz berries (Rogiers et al., 2004), is in line with our microscopic analyses (**Figure 7**) and the repression of cuticle and wax biosynthetic genes in L15 (**Figure 6**). With the beginning of berry ripening, the photosynthetic apparatus is dismantled and consequently the photosynthetic capacity of the berry drops dramatically (Pandey and Farmahan, 1977). *VviERF045* could contribute to this switch-off in virtue of its effect on chlorophylls and carotenoids content (**Figure 3**), and the down-regulation of genes important for chlorophyll accumulation, like *CIP7* and *FERRITINs*.

Many pathogen-resistance genes appear modulated by *VviERF045* (Supplementary Table S4), suggesting its action also increases plant defense via activation of the basic immune defense system. Among the proteins that change their levels of expression during berry ripening, there are many pathogenesis-related proteins (PRs). PRs are highly abundant at ripening and generally lowly expressed or absent in unripe berries. The presence of this class of proteins in healthy fruit suggests that they may play a role in fruit development, or that they are part of a pre-emptive defense when softening and sugar accumulation make fruit attractive targets for pathogens (Davies and Robinson, 2000).

To further corroborate the importance of the obtained results in understanding berry ripening regulation, we ran *in silico* analyses taking advantage of the grapevine gene expression compendium VESPUCCI (Moretto et al., 2016). We looked whether the 563 DEGs modulated in the L15 to WT plants comparison, were expressed in the berry during ripening, in order to gain insights about their role in the process. Five hundred and forty five DEGs (18 genes were not unique in the database) were analyzed in 389 condition contrasts (Supplementary Table S5) mostly derived from samples of berries at different phenological stages, between EL 27 and EL 41. Interestingly, a large fraction (70%) of the DE genes appeared either up- (153 genes) or down-regulated (231 genes) (Supplementary Table S4), indicating that these genes are indeed modulated during ripening. The two groups were also enriched in functional classes characteristic of berry ripening like starch and sucrose metabolism, auxin biosynthesis, ethylene signaling and phenylpropanoid biosynthesis in the case of the up-regulated genes, cell wall and HomeoBox TFs in the case of the down-regulated ones. An important interaction between ethylene and auxin in the control of berry ripening has been recently elucidated (Böttcher et al., 2013). Within the DEGs, we found 7 *ERF* encoding genes: two *SHINE* putative horthologues (*VviERF042* and *VviERF044*) that were down-regulated, and other five *ERF*s that were strongly up-regulated. In this last group with the exception of *VviERF045*, there were four *ERF* genes (*VviERF093, VviERF111, VviERF118, VviERF120*), from clade IX or X, previously shown to be induced in the transition from véraison to ripe berries either in skin or in flesh (Licausi et al., 2010). These evidences strongly suggest an involvement of these ERF TFs in the control of berry ripening.

## Conclusion

We have functionally characterized *VviERF045* by overexpressing the encoding gene in *in vitro* grown grapevine plants and by phenotyping them at morphological and molecular level. *VviERF045* seems to regulate, in coordination with other ERF factors, including the putative horthologues of the *Arabidopsis SHINE1* and *3* genes, different processes such as the structuring of the epidermis and cuticle of the berry, cell expansion, photosynthesis, phenylpropanoid metabolism and the activation of several defense related genes. If this functional role will be confirmed by follow-up studies on the fruits of the transgenic lines, we can predict that having the possibility to adjust the expression of *VviERF045* by well-timed viticultural practices (e.g. water stress, hormonal treatments) or by breeding, might allow to improve grape quality and plant resilience. The expression of *VviERF045* can be used as an expression marker of the plant resilience status.

## Author Contributions

CL, AD, VP, and DM did the experimental work, CL, LD, MG, and GR assessed the best way to prove the gene function, CL did the phylogenetic trees, PS and KE elaborated RNA-seq data and were involved in data interpretation, CL, GR, and CM substantially contributed to the design of the work. All the authors revised it critically for important intellectual content and approved the final version of this manuscript.

## Conflict of Interest Statement

The authors declare that the research was conducted in the absence of any commercial or financial relationships that could be construed as a potential conflict of interest.

## Footnotes

1 http://www.bioinformatics.bbsrc.ac.uk/projects/fastqc

2 https://ccb.jhu.edu/software/fqtrim/index.shtml

3 http://genomes.cribi.unipd.it/grape/

4 http://www.ncbi.nlm.nih.gov/geo/

5 www.R-project.org/

## Supplementary Material

FIGURE S1 **Library size of each RNA-seq sample replicate**.

FIGURE S2 **Comparison of RNA-seq and real time RT-qPCR analysis.** Expression profiles of *VviERF045 VIT_04s0008g06000, RPT2 VIT_00s0665g00050, PIR VIT_00s1306g00010, NAC VIT_01s0026g02710, ERECTA VIT_04s0008g01970, SBP VIT_04s0210g00170, N-acetyltransferase VIT_05s0020g03680, HB1 VIT_12s0059g01190, SA VIT_14s0006g02170, YABBY VIT_15s0048g00550, Peroxidase VIT_16s0098g00820, HB1 VIT_18s0001g10160*. Lines represent expression levels (log Fold Change) by RNA-seq analyses in WT and L15, reported as means and standard errors of three independent biological replicates. Histograms represent the relative expression levels (logFC) to the expression of the WT, as assessed by real time RT-qPCR and reported as means and standard errors of three biological and two technical replicates for each plant line. Different letters show significant differences among samples with *p* < 0.05 and Tuckey’s significance test. In case of no significance no letters are reported in the figure.

FIGURE S3 **GO term enrichment in selected DEGs. (A)** Blast2GO Fisher’s enrichment test analysis for GOterms. Blue bars indicate Test Set (DEGs from L15 vs. WT comparison) while red line indicate the Reference Set (entire reference transcriptome). On the *X*-axis is reported the percentage of sequences for each GO category **(B)** Best 9 GOterms by comparing classic with weight method from Fisher’s test elaborated with TopGO (Alexa et al., 2006).

FIGURE S4 **PCA score and loading plots of metabolites in the transgenic lines.** Different color points represents different samples: black (L15), red (L19), green (L22), blue (L6), light blue (L7), pink (WT). Distribution of the average values in **(A)** all the analyzed metabolites, **(B)** phenolic compounds, **(C)** anthocyanins **(D)** lipids **(E)** chlorophylls (Ca and Cb coincide) and total carotenoids (Cxc). The most weighted loadings are represented in each plot.

FIGURE S5 **Transcripts involved in Terpene biosynthesis.** 4.2.3.46 alpha-farnesene synthase (AFS1) (*VIT_00s0361g00060, VIT_00s0392g00030, VIT_00s0392g00060*), 1.14.99.7 (*VIT_00s0441g00020*) squalene monoxygenase, 4.2.3.13 (*VIT_18s0001g04710*) (+)-delta-cadinene synthase, 4.2.3.75 (-)-germacrene D synthase (*VIT_18s0001g04990, VIT_18s0001g05240*), 4.2.3.119 (*VIT_08s0007g06860*) pinene synthase, 1.14.-.- CYP82C4 (*VIT_18s0001g11480*), 1.3.3.9 CYP72A1 secologanin synthase (*VIT_19s0135g00150*), 1.14.13.72 C-4 sterol methyl oxidase (*VIT_00s2125g00010*), CYP724B1 (*VIT_14s0066g00170*), CYP90B1 Steroid 22-alpha-hydroxylase (*VIT_04s0023g01630, VIT_04s0023g01640, VIT_12s0057g01460*). Green color means down-regulated gene, red color means up-regulated gene.

FIGURE S6 **Transcripts involved in alpha-linolenic metabolism.** 1.13.11.12 LOX1 (*VIT_06s0004g01470*) lipoxygenase 1, 21.1.141 Jasmonate *O*-methyltransferase *(VIT_14s0006g02170)*, MJAE MeJA esterase *(VIT_00s0253g00090).* Red color means up-regulated gene.
