# Supplementary figures and images for: Insights into the Role of the Berry-Specific Ethylene Responsive Factor VviERF045
Source: Front Plant Sci. 2016 Dec 9;7:1793. doi: 10.3389/fpls.2016.01793 (PMC5146979; doi:10.3389/fpls.2016.01793)

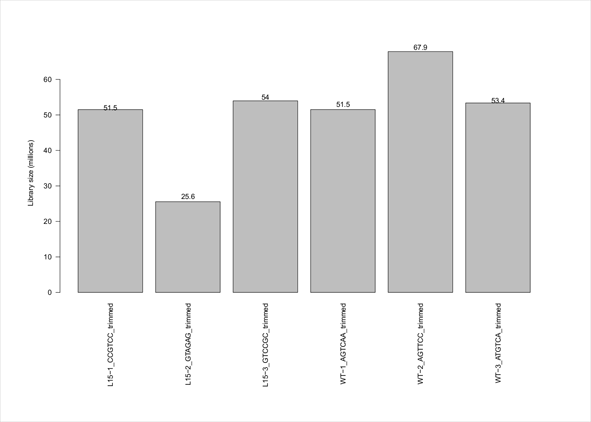

Supplement: FIGURE S1 — Library size of each RNA-seq sample replicate. [file Image_1.TIF]

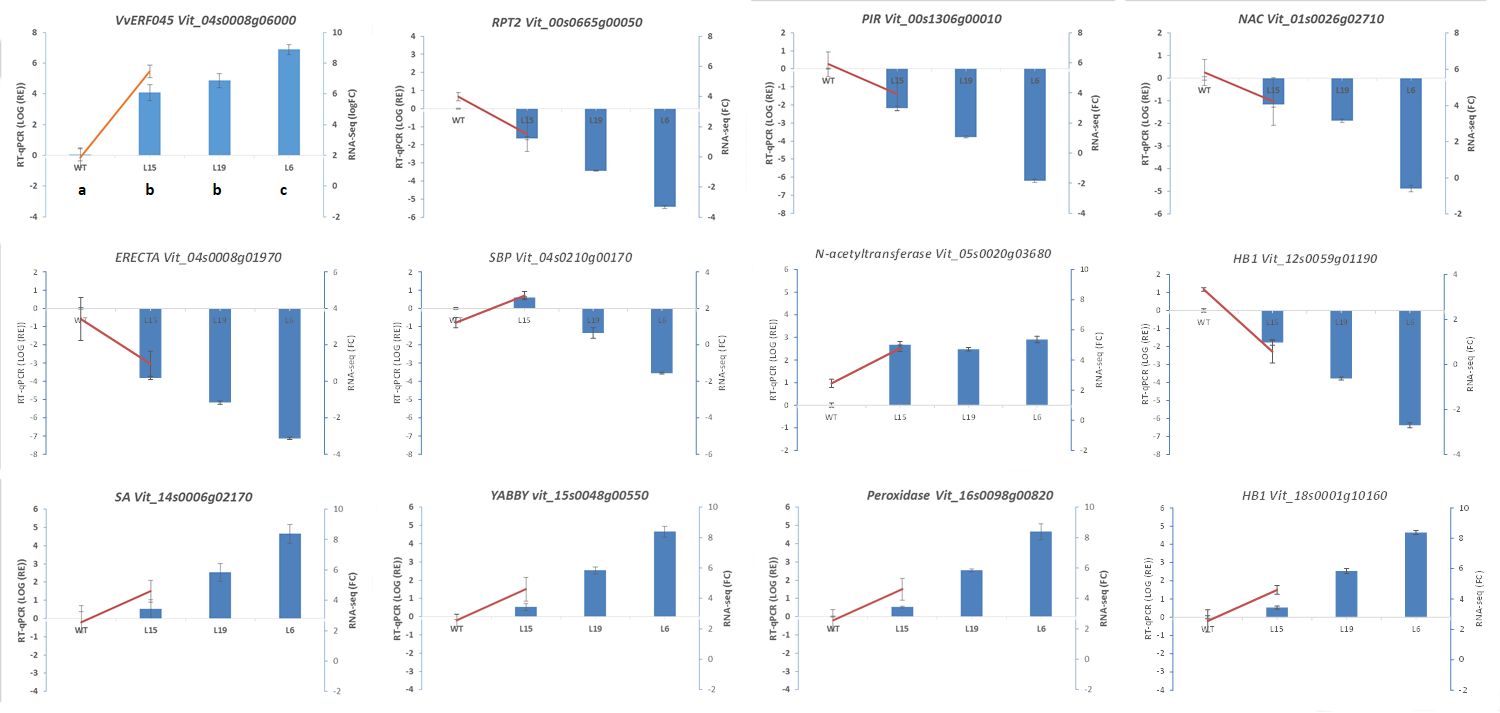

Supplement: FIGURE S2 — Comparison of RNA-seq and real time RT-qPCR analysis. Expression profiles of VviERF045 VIT_04s0008g06000, RPT2 VIT_00s0665g00050, PIR VIT_00s1306g00010, NAC VIT_01s0026g02710, ERECTA VIT_04s0008g01970, SBP VIT_04s0210g00170, N-acetyltransferase VIT_05s0020g03680, HB1 VIT_12s0059g01190, SA VIT_14s0006g02170, YABBY VIT_15s0048g00550, Peroxidase VIT_16s0098g00820, HB1 VIT_18s0001g10160. Lines represent expression levels (log Fold Change) by RNA-seq analyses in WT and L15, reported as means and standard errors of three independent biological replicates. Histograms represent the relative expression levels (logFC) to the expression of the WT, as assessed by real time RT-qPCR and reported as means and standard errors of three biological and two technical replicates for each plant line. Different letters show significant differences among samples with p < 0.05 and Tuckey’s significance test. In case of no significance no letters are reported in the figure. [file Image_2.TIF]

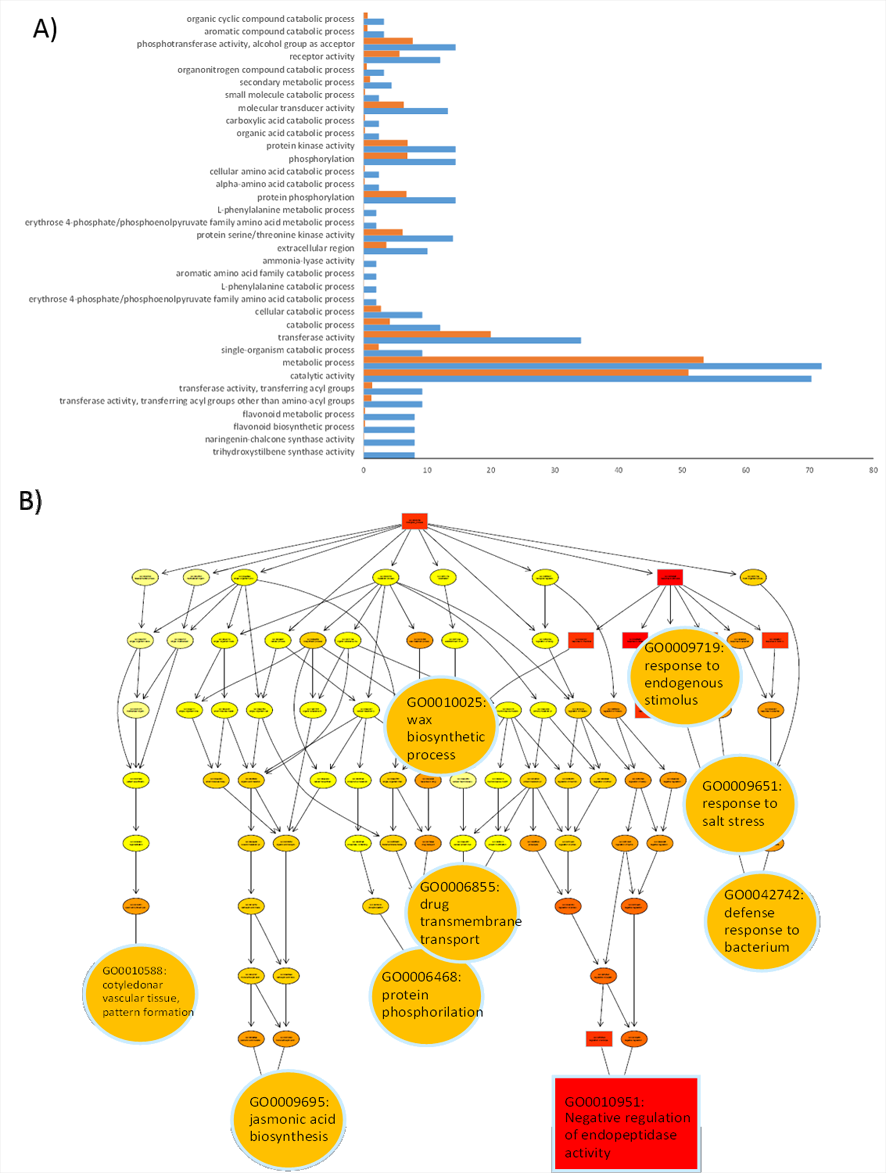

Supplement: FIGURE S3 — GO term enrichment in selected DEGs. (A) Blast2GO Fisher’s enrichment test analysis for GOterms. Blue bars indicate Test Set (DEGs from L15 vs. WT comparison) while red line indicate the Reference Set (entire reference transcriptome). On the X-axis is reported the percentage of sequences for each GO category (B) Best 9 GOterms by comparing classic with weight method from Fisher’s test elaborated with TopGO (Alexa et al., 2006). [file Image_3.TIF]

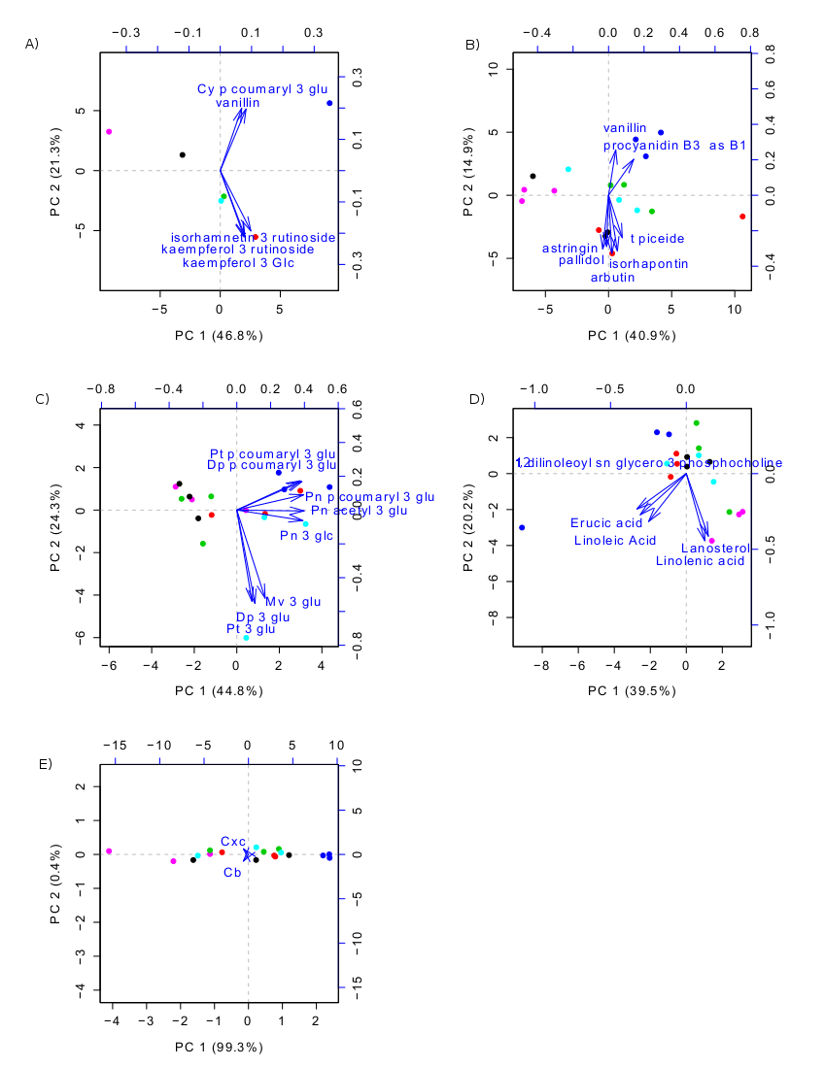

Supplement: FIGURE S4 — PCA score and loading plots of metabolites in the transgenic lines. Different color points represents different samples: black (L15), red (L19), green (L22), blue (L6), light blue (L7), pink (WT). Distribution of the average values in (A) all the analyzed metabolites, (B) phenolic compounds, (C) anthocyanins (D) lipids (E) chlorophylls (Ca and Cb coincide) and total carotenoids (Cxc). The most weighted loadings are represented in each plot. [file Image_4.TIF]

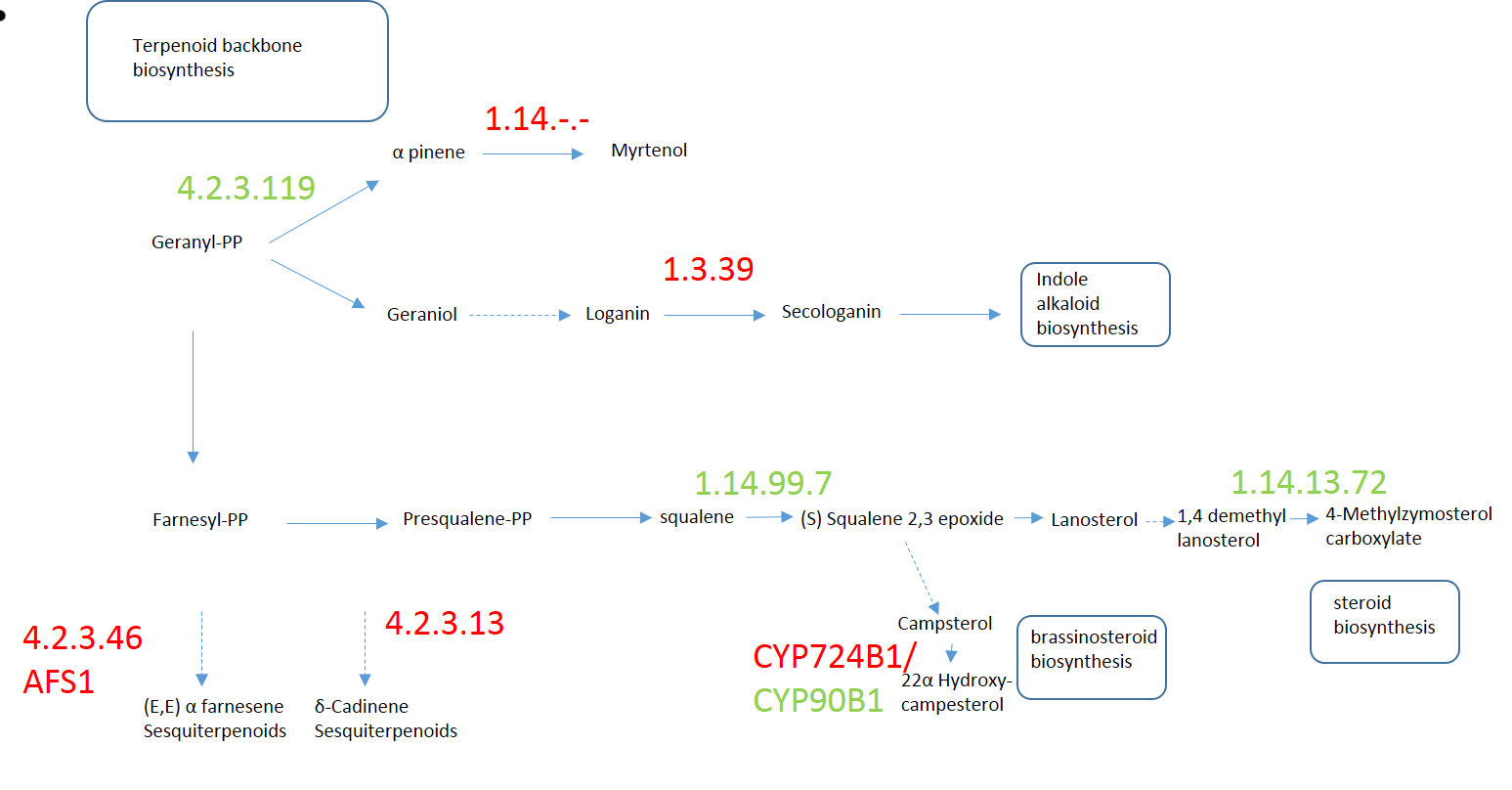

Supplement: FIGURE S5 — Transcripts involved in Terpene biosynthesis. 4.2.3.46 alpha-farnesene synthase (AFS1) (VIT_00s0361g00060, VIT_00s0392g00030, VIT_00s0392g00060), 1.14.99.7 (VIT_00s0441g00020) squalene monoxygenase, 4.2.3.13 (VIT_18s0001g04710) (+)-delta-cadinene synthase, 4.2.3.75 (-)-germacrene D synthase (VIT_18s0001g04990, VIT_18s0001g05240), 4.2.3.119 (VIT_08s0007g06860) pinene synthase, 1.14.-.- CYP82C4 (VIT_18s0001g11480), 1.3.3.9 CYP72A1 secologanin synthase (VIT_19s0135g00150), 1.14.13.72 C-4 sterol methyl oxidase (VIT_00s2125g00010), CYP724B1 (VIT_14s0066g00170), CYP90B1 Steroid 22-alpha-hydroxylase (VIT_04s0023g01630, VIT_04s0023g01640, VIT_12s0057g01460). Green color means down-regulated gene, red color means up-regulated gene. [file Image_5.TIF]

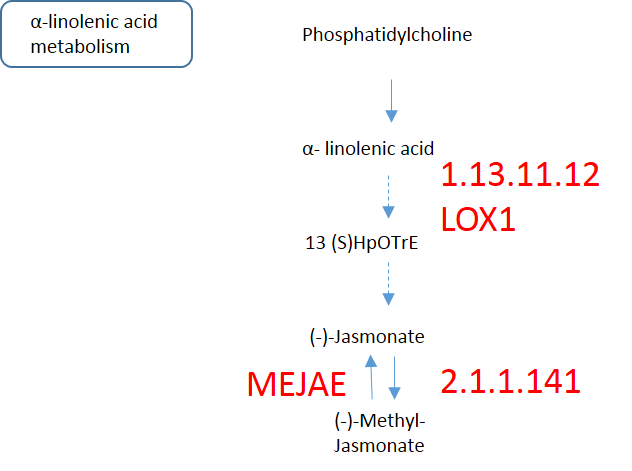

Supplement: FIGURE S6 — Transcripts involved in alpha-linolenic metabolism. 1.13.11.12 LOX1 (VIT_06s0004g01470) lipoxygenase 1, 21.1.141 Jasmonate O-methyltransferase (VIT_14s0006g02170), MJAE MeJA esterase (VIT_00s0253g00090). Red color means up-regulated gene. [file Image_6.TIF]
